# Smoking Cessation Counseling in Practice: A Qualitative Analysis of Quitline Conversations in Queensland, Australia

**DOI:** 10.1177/10901981231206068

**Published:** 2023-10-17

**Authors:** Hollie Bendotti, Sheleigh Lawler, Coral Gartner, David Ireland, Henry M. Marshall

**Affiliations:** 1 Thoracic Research Centre, Faculty of Medicine, The University of Queensland, Chermside, Australia; 2 The Australian e-Health Research Centre, Commonwealth Scientific and Industrial Research Organisation, Herston, Queensland, Australia; 3 School of Public Health, The University of Queensland, Herston, Australia; 4 NHMRC Centre of Research Excellence on Achieving the Tobacco Endgame, School of Public Health, Faculty of Medicine, The University of Queensland, Herston, Australia; 5 The Prince Charles Hospital, Metro North Hospital and Health Service, Chermside, Queensland, Australia

**Keywords:** smoking cessation, Quitline, tobacco, behavior change, counseling

## Abstract

Telephone-based services are a practical and effective behavioral support for smoking cessation, yet no in-depth analyses of this counseling have been conducted. Understanding the general content of Quitline conversations can help to improve current practices and may inform future interventions. Therefore, we aimed to independently explore conversation themes, topics, and client questions during Quitline counseling sessions with Quitline clients in Queensland, Australia. A purposive sample of 30 recorded counseling sessions, completed between January and March 2019, were de-identified, transcribed, and thematically analyzed. Seven themes, encompassing 35 topics, were derived from 26 initial calls and four follow-up calls: (1) Client details and building rapport; (2) Client history and motivation to quit; (3) Pharmacotherapy; (4) Behavioral aspects of quitting and relationship with smoking; (5) Understanding nicotine dependence and other important considerations; (6) Additional support and smoking cessation resources; and (7) Planning, goal setting and follow-up. Three themes emerged from 18 client questions including (1) Pharmacotherapy safety and contraindications; (2) Pharmacotherapy instructions and mechanism of action; and (3) Physiology of nicotine dependence. This is the first qualitative analysis of the content of Quitline counseling sessions in Australia. Counselors collect and deliver a breadth of information to provide tailored, evidence-based health care, while building rapport and trust. Findings may be translatable into personalized self-help interventions that are more accessible or appealing to people reluctant to contact Quitline. Harnessing educational opportunities regarding pharmacotherapy adherence and misconceptions can improve client confidence in the product and smoking cessation outcomes. Further research will map conversations to motivational interviewing and behavior change techniques.

Smoking is the leading behavioral risk factor contributing to global burden of disease (Stanaway et al., 2018), and more than eight million premature deaths are attributable to tobacco use alone every year worldwide (World Health Organization, 2017). Tobacco dependence is a chronic relapsing-remitting condition for many people who smoke (Steinberg et al., 2008), and successful cessation normally requires multiple quit attempts (Chaiton et al., 2016). It is well-known that quitting smoking has significant positive short- and long-term health outcomes for all ages, therefore acceptable, accessible, and effective smoking cessation support that increases the likelihood of permanent smoking cessation is important to reducing tobacco-related disease burden.

People who are motivated to quit smoking may benefit from intensive behavioral support in the form of counseling. Multiple styles of counseling have been deemed appropriate and/or effective for smoking cessation including cognitive behavioral therapy (CBT; Marks & Sykes, 2002; Sykes & Marks, 2001; Vinci, 2020), motivational interviewing (MI; Lindson-Hawley et al., 2015), acceptance and commitment therapy (Gifford et al., 2004), and mindfulness (de Souza et al., 2015; Vinci, 2020). Telephone-based services, such as Quitline, are a form of personalized behavioral counseling that can both substitute and supplement other forms of smoking cessation interventions, but are of lower-cost and greater reach than face-to-face counseling services. Quitline counselors are trained to primarily deliver CBT strategies and/or MI alongside evidence-based information relative to all stages of the quit journey (World Health Organization, 2014). These services can operate reactively by providing advice and support to people who initiate contact with the Quitline, or proactively, whereby counselors make contact to provide support during a quit attempt through a referral system (Matkin et al., 2019). Proactive telephone counseling and multiple support calls have been shown to increase the chances of quitting and quit rates (Matkin et al., 2019), respectively.

Quitline services have been increasingly adopted around the globe after the very first dedicated smoking cessation telephone line was introduced in Australia by Quit Victoria in 1985 (Anderson & Zhu, 2007). Today, the Australian Quitline service is funded by each state and territory, with varying administrative and contractual arrangements (Greenhalgh et al., 2020). There were no national standards at the time of our data collection; however, National Quitline Minimum Standards have now been published to ensure consistency in service delivery across states and territories (Cancer Council Victoria, 2021). In Queensland, Quitline is funded by the State Government (Queensland Health). All clients receive an initial call to formulate a quit plan and an ad hoc follow-up schedule, while some individuals are eligible for specific programs and receive three scheduled support calls to review progress. The Queensland Quitline is unique in Australia as it is the only Quitline service that provides free nicotine replacement therapy (NRT) to specific priority groups, including Aboriginal and Torres Strait Islander peoples, regional/remote populations, and pregnant women and people under the care of community mental health teams (Queensland Health, 2022). Australian Quitlines have previously been rated highly by those accessing these services, and the majority of callers sampled in evaluation studies have made a quit attempt at various time periods of follow-up (Hayes et al., 2012; Wakefield & Miller, 1999). Moreover, higher quit rates were observed in states which employed a proactive call-back service compared with those states which did not (Borland et al., 2001). Quitlines also provide a high degree of equity of access, as well as dedicated programs for the aforementioned priority groups as well as those experiencing mental illness and people who have been incarcerated (Greenhalgh et al., 2020). However, only 2% of Australians who smoke daily and made a quit attempt in the past year indicated they accessed Quitline (Australian Institute of Health and Welfare, 2020), highlighting a potential need for strategies to improve current uptake or translate these services into more acceptable platforms.

Quitline counseling sessions provide practical and effective (Matkin et al., 2019) tailored behavioral support for smoking cessation, yet there is a paucity of research utilizing this data. Previous articles have described telephone counseling protocols in the United States (Tedeschi et al., 2005; Zhu et al., 1996). In addition, a single study assessed the fidelity of telephone behavioral support in the United Kingdom by comparing the prevalence of behavior change techniques (BCTs) in telephone sessions with those identified in training manuals. They found that overall fidelity was low, with variability between counselors and type of session, and that counselors perceived they had used more BCTs than they actually did (Lorencatto et al., 2014). However, to-date there has been no independent qualitative analysis of the general content within Quitline counseling sessions, or in the Australian context. Understanding the common conversation topics and themes from lived experiences within Quitline calls may help to strengthen current practice and inform future smoking cessation interventions, elucidate misconceptions and promote the uptake of Quitline services, and assist in educating/training health professionals in the delivery of smoking cessation advice. Therefore, this study aims to explore the content (conversation themes, topics, and client questions) of Quitline counseling sessions with people who want to quit tobacco smoking in Queensland, Australia.

## Method

### Study Design

This qualitative study used a sample of recorded telephone counseling sessions from an administrative dataset provided by Quitline (Queensland Health). All Quitline calls are recorded for quality assurance and training. In Queensland, over 87% of Quitline counselors have tertiary qualifications in counseling, psychology, or social work, while the remaining counselors either have a diploma qualification or are actively working toward a relevant tertiary qualification. Regardless of previous qualifications, all Quitline counselors undergo specific standardized smoking cessation onboarding training, monitoring, and continuing professional development. Sample transcripts were used for thematic analysis of conversation topics and themes, and to identify questions asked by clients during the calls.

### Ethical Considerations

This study was approved by The Prince Charles Hospital Human Research Ethics Committee (Project ID: 50620). A formal agreement between HM and Quitline, and waiver of obtaining individual informed consent via a Public Health Act 2005 (PHA 50620) application were obtained prior to accessing the audio files.

### Sampling of Quitline Sessions and Data Collection

Purposive sampling of 30 audio files was independently completed by Quitline based on demographic characteristics including age, sex, rural/remote location, and Indigenous status. This was to ensure diversity in participants and experiences. Audio files were manually transcribed verbatim and identifiable information was redacted prior to analysis. Calls were completed between January and March 2019, and call length ranged from 16 to 56 min (Mean = 36 min, *SD* 10 min).

### Thematic Analysis

Individual files were imported to NVivo (Version 12), and coded topics were also transferred to and collated in sequence in Microsoft Excel for each transcript. Thematic analysis followed Braun and Clarke’s six-phase framework (Braun & Clarke, 2006), and was completed by two study team members (senior researcher and early career researcher) with formal training and experience in qualitative research as well as expertise in health psychology, health promotion and public health. Team members had not viewed any published protocols, guidelines or standards pertaining to the Quitline counseling to allow for an independent analysis and to minimize the risk of bias in theme and topic development. Together, both researchers (H.B. and S.L.) inductively coded a simple random sample of five transcripts to determine an initial set of topics. The remaining transcripts were then coded, and initial sample re-coded, by one researcher (H.B.) by manual examination and classification. New topics were created for those that did not fit with existing codes and were discussed and agreed upon by both researchers (H.B. and S.L.). Following coding of all transcripts, the main themes were defined by grouping topics, and were reviewed and refined by both researchers. All disagreements regarding interpretation of topics and themes were able to be resolved via discussion between the two study members, but a third team member (senior researcher) was available for consensus if required (H.M.) and has expertise in thoracic medicine, psychology, and smoking cessation. Questions asked by clients were collected separately, and main themes were defined, discussed, and agreed upon by both researchers (H.B. and S.L.).

## Results

Thirty transcripts from 30 individuals were analyzed, of which 26 were initial calls and four were support calls. Calls were completed by 23 individual counselors, of which almost all were female (*n* = 21). Most clients were female (*n* = 16), resided in a regional/rural area (*n* = 19) and the mean age was 44.3 years (*n* = 29). The majority of initial calls (85%) were initiated by Quitline. Referral source information was available for 15 clients and included hospital (*n* = 5) and community health (*n* = 5) services, workplace programs (*n* = 4), and one self-referral (i.e., requested a call via website or telephone).

At the initial call, three clients (12%) self-reported as being abstinent from smoking. Of those who discussed methods used for previous quit attempts (*n* = 27), nine clients (33%) reported no previous use of evidence-based cessation pharmacotherapy products (i.e., tried to quit “cold turkey” or only used an alternative therapy), 11 (41%) had tried more than one type of product, and seven (26%) had only used one product. The quit plans discussed always incorporated a combination of pharmacotherapy and behavioral strategies. Combination nicotine replacement therapy (NRT) was discussed and recommended as the primary quit method for more than two-thirds of clients during their initial call (*n* = 19). Two clients were also still using combination NRT at their support call. See Table 1 for descriptive summary of calls and Supplementary Table 1 for descriptive summaries of individual transcripts.

**Table 1.** Summary Characteristics of Quitline Calls.

	*n*	*M* (*SD*)	Minimum	Maximum
**Call length (min:s)**	30	35:59 (10:09)	15:59	56:18
**Type of call**				
Initial	26			
Support	4			
**Type of initiation (initial calls only)**				
Proactive (by Quitline)	22			
Reactive (by client)	4			
**Client demographics**				
Age (years)	29	44.3 (13.4)	19	66
Gender				
Male	14			
Female	16			
Location				
Metropolitan	11			
Regional/rural	19			
Indigenous status				
Non-indigenous	9			
Indigenous	3			
Unknown	18			
**Smoking and cessation behavior**				
Smoking status				
Current	23^a^			
Current but cutting down	3^b^			
Quit	4^c^			
Cigarettes per day (pre-quit)	27	16.5 (6.9)	7	30
Age commenced smoking (years)	23	16.2 (3.9)	7	28
Length smoking (years)	22	27.9 (13.6)	1	49
Previous quit attempt (initial calls only)				
Yes	26			
No	—			
Longest quit attempt	24		3 days	5 years
**Quit plan**				
Follow-up discussed				
Yes	25			
No	5^d^			
Quit date set				
Yes	9			
No	17^e^			
NA (i.e., Quit)	4			
Method discussed				
Combination NRT	21			
NRT patch	2			
Oral NRT	4^f^			
Varenicline	3			
Bupropion	1^f^			

*Note*. NRT = nicotine replacement therapy.

a 21 initial calls; two support calls. ^b^One initial call; two support calls. ^c^Three initial calls; one support call. ^d^One incomplete audio file; one final call of Quitline program. ^e^One incomplete audio file. ^f^Combined bupropion and oral NRT.

### Conversation Themes and Topics

**Figure 1.**
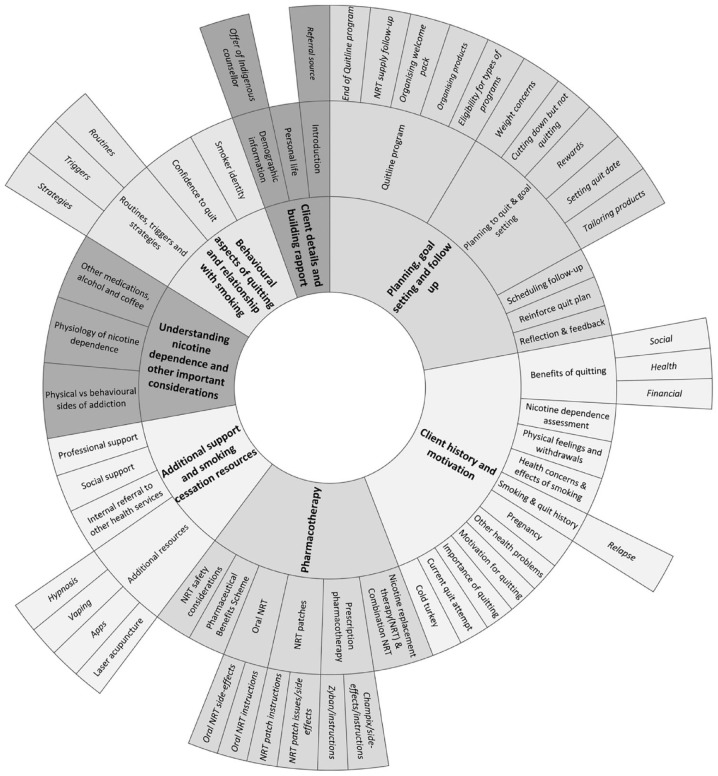
Conversation Themes and Topics.

Seven themes, encompassing 35 topics, were identified across the transcripts (Figure 1). Supplementary Table 2 describes topics included under relevant themes.

#### Client Details and Building Rapport

Quitline counselors established rapport with their clients through greetings, obtaining consent and checking personal details at the outset of all conversations. Counselors strengthened rapport throughout the counseling session using active listening skills (i.e., listening, understanding, and verbally reflecting on what was said) and positive feedback during discussions around clients’ history and personal lives:

. . . that’s a massive achievement and from having . . . such a traumatic time all those years ago to . . . finishing your book. [Counselor, T5]

Standard demographic information, including date of birth and postcode, was initially collected and requested at support calls to confirm identity. Clients who identified as Aboriginal and/or Torres Strait Islander were always offered the option of an Indigenous counselor.

#### Client History and Motivation to Quit

Smoking and quit histories, including active quit attempts, were always thoroughly explored to develop and personalize quit plans. These included duration of smoking, reasons for initially starting smoking, experiences with withdrawals and quitting “cold turkey,” changes in smoking behavior, previous experiences with pharmacotherapies and quitting in general, including reasons for relapse:

Is cold turkey the only way that you’ve tried to do it before? [Counselor, T20]

Specific questions regarding length of use of pharmacotherapy allowed for education of optimal treatment course length when previous length of use was insufficient:

Counselor: “. . . did you use full 12 weeks of the patches?”

Client: “No just for 1 week.”

Counselor: “Oh, you only used it for 1 week?”

Client: “Yeah.”

Counselor: “Okay yeah. So, evidence shows that it’s best to use the patches for about 8 to 12 weeks since your last cigarette.” [T4]

Nicotine dependence assessment (cigarettes per day and time to first cigarette after waking) was used to guide level of pharmacotherapy support, and clients were also sometimes asked if they agreed that they had a level of dependency.

Discussions around personal reasons and motivations to quit were observed in most conversations, and allowed for exploration of anticipated and felt benefits of quitting, including financial, health and social benefits. Health issues, conditions and concerns directly related to smoking were occasionally shared by clients, as well as other health problems that provided a teachable moment. At times, these were an opportunity for counselors to build rapport through empathy, and boost self-efficacy by highlighting successful behavioral strategies previously employed for health issues that could be applied in the context of smoking cessation:

Client: “I just recently had a massive heart attack.”

Counselor: “Hmm okay. Horrible, sorry to hear.”

Client: “Yeah I was in hospital for 10 days so I want to give up but I struggle with it.” [T11]

“I’m wondering when you say you gave up alcohol . . . How did you do that? What strengths did you draw on that you might be able to apply for giving up smoking?” [Counselor, T27]

Finally, importance of quitting smoking to the client was occasionally gauged using a 10-point scale toward the end of conversations or prompted directly by counselors to encourage client reflection.

#### Understanding Nicotine Dependence and Other Important Considerations

Some counselors educated clients on the two sides of addiction (physical versus behavioral). Discussions around client history, more specifically their experience with relapse and withdrawal, allowed for teachable moments regarding the physiology of nicotine dependence, such as reassurance that the withdrawal symptoms would lessen over time. This education also assisted clients to understand the mechanism of action of pharmacotherapy, in turn making it easier for counselors address any concerns the clients had about the pharmacotherapies:

Those receptors in your brain that respond to the nicotine, they never actually die off or disappear, but after about 12 weeks of not smoking at all, they do become dormant. [Counselor, T26]

Similarly, teachable moments regarding reducing coffee and alcohol intake when quitting were also presented when developing associated behavioral strategies, as well as advising clients to notify their general practitioner (GP) that they were quitting smoking so that any medications they were on could be reviewed and their medical records updated.

#### Pharmacotherapy

The use of pharmacotherapy to quit smoking was recommended across all conversations. Counselors educated clients on the mechanism of action for NRT products (including combination NRT) and prescription pharmacotherapies (varenicline and bupropion), while also providing instructions for use, and advising of potential side-effects and associated solutions. Instructions for use of prescription pharmacotherapies was limited to general treatment course duration as counselors advised clients to speak to their GP for more personalized advice concerning these medicines. Moreover, some clients were encouraged to access subsidized NRT products via a GP under the Pharmaceutical Benefits Scheme if they were not eligible for free products through Quitline. Clients who were eligible for, and wanted, NRT from Quitline were asked a series of medicine safety questions related to recent cardiac events, pregnancy, and skin conditions, before dispatch of products.

#### Behavioral Aspects of Quitting and Relationship With Smoking

The behavioral aspects of quitting were frequently explored and reinforced. Counselors assisted clients in recognizing individual routines and triggers through direct open-ended questions, or through discussions about their smoking and quitting history, or personal lives:

Counselor: “What returned you to smoking would you say?”

Client: “Everybody around me smoked.”

Counselor: “So being around other smokers.” [T21]

Management strategies for triggers were often personalized and developed in partnership between the counselor and client, drawing upon generic strategies and client preferences. Clients’ level of confidence to quit was typically gauged using a 10-point scale toward the end of the conversation, which gave counselors the opportunity to give positive reinforcement or suggest further strategies or tools to help improve low confidence:

Counselor: “Yeah so with those strategies in mind, how confident do you feel that you’re able to quit from this Thursday?”

Client: “Um well actually after talking to you, a lot more confident that I thought I’d be.”

Counselor: “Yeah? Okay that’s great. So, if you had to rate it out of 10.”

Client: “Oh about 9, 9 and a half.” [T4]

Counselors also directly and indirectly encouraged clients to better understand their own identity, attitudes, and personal relationship with smoking to elicit further motivation for behavioral change:

Counselor: I mean if you were to look at the reasons of what you enjoy about smoking what would those things be?

Client: To be honest, no, nothing. I actually know it’s a habit. I actually sit there smoking one thinking why, I don’t even want it. [T21]

#### Additional Support and Smoking Cessation Resources

Counselors often enquired about other avenues of support for clients, helping them to identify their social support networks and explore perceived levels of support. This also included developing management strategies for those clients who lived with, or spent time with, other people who smoked (e.g., partners or friends):

Who have you got as your cheer squad with quitting? [Counselor, T14]

Counselors promoted accessing other health care professional support for quitting, such as GPs, and encouraged clients to consider accessing carer support and mental health services if appropriate after learning more about their individual circumstances. Furthermore, internal referrals to partner public health services, and their associated smoking cessation programs, were offered to clients with specific health conditions as additional support.

Alternative treatments and resources for smoking cessation were discussed when exploring quit histories and developing quit plans. Some clients indicated they had tried hypnosis, and laser acupuncture in past quit attempts. Others had tried, or had known someone who had tried vaping, or had questions regarding vaping specifically. Responses to such questions reinforced use of guideline-approved therapies, and counselors were conscious of not recommending methods which lacked a strong supporting evidence base. Conversely, free smartphone apps for smoking cessation were occasionally promoted to help client’s track aspects of the quit journey (e.g., money saved, health improvements, and cigarettes not smoked) as a means of motivation and encouragement.

#### Planning, Goal Setting and Follow-Up

Elements of the Quitline program were described in detail throughout conversations to inform individual quit plans. Clients were advised of the specific programs available to them regarding number of follow-up calls and their eligibility for free NRT. Arrangements for delivery of information packs and/or NRT products were made during initial calls, and NRT supplies reviewed at support calls. Pharmacotherapy products sometimes needed to be tailored due to health conditions or previous side-effects.

Quit plans were always developed in partnership, with agreed upon pharmacotherapy and behavioral strategies guided by a culmination of smoking and personal history, previous quitting experience, and individual motivations and goals relative to quitting, health and personal life. Some clients were asked to consider setting a quit date, including whether the selected date had any personal significance, and how they may reward themselves for achieving goals:

Counselor: “So, in terms of quitting smoking, did you have an idea when you would like to be doing that?”

Client: “That would be this coming Thursday.”

Counselor: “Is there anything important about that date?”

Client: “That’s when I get paid and I can afford to buy some patches”

Counselor: “Okay great. That is a very important day. Pay day is an important day” [T4]

Toward the end of calls, counselors would sometimes ask for client feedback and encourage them to reflect on the most helpful elements for their quit attempt, while also frequently reinforcing quit plans and scheduling follow-up support calls:

It’s been good to talk to somebody and kind of talk through the whole thing . . . As I’ve said like, I want to give up smoking but it’s just so hard. I just find it so hard to do. So, it’s actually good to talk to someone about it. [Client, T11]

### Client Questions Themes

Eighteen questions were collected from 12 conversations (10 initial; two support). Table 2 outlines the theme structure with associated questions.

**Table 2:** Themes Associated With Questions Asked by Clients.

Main theme	Subtheme	Question
Pharmacotherapy safety concerns & contraindications	Oral NRT	“And another concern I had was like would those cause anything in the mouth or tongue cancer or?”
		“An inhalator. What’s it got in it?” (Question in relation to friend of client who became addicted to NRT inhalator)
		“Can you OD?”
	NRT Patches	“But you wouldn’t use them (patches) with Champix would you?”
		“You don’t overdose on them?” (double patching)
		“I’m on the anti-depression medication. So, does the patches affect in anyway?”
		“So you will get addicted to the patches then?”
	Varenicline	“So being on Champix for a while, is it harmful?”
Pharmacotherapy instructions and mechanism of action	Oral NRT	“How does the spray work?”
	NRT patches	“So you wear them to bed?”
		“I read something on the packet the other day that patches “should be kept at 24 degrees”. So I think should I keep them in the fridge or is that going to make any difference?”
		“Can you have a patch on and take the lozenges or no? Is that bad?”
		“Well my, if I take like for example, if I say put the patch on or whatever. And someone is smoking in the car, will it make me want to have a smoke?”
		“Oh true, not in the morning?” (in response to “. . . put them on in the evening”)
		“So you obviously take them (patches) off while you’re smoking yeah?”
		“So the patches and that, does that actually have the nicotine in them?”
Physiology of nicotine dependence	—	“So being on the nicotine replacement, wouldn’t those neuroreceptors still be getting reignited from the lozenges and the patches anyway? Like they’re getting the nicotine?”
		“Is it the nicotine that does that (stress response) or all the other s**t that’s in the smoke?”

NRT = nicotine replacement therapy.

#### Pharmacotherapy Safety Concerns and Contraindications

Some clients expressed concerns regarding the potential for overdosing on NRT, addiction to NRT, and long-term harms of different pharmacotherapies, including any possible carcinogenic effects. Interactions between NRT patches and other prescription medications were queried, as well as the use of varenicline alongside NRT patches.

#### Pharmacotherapy Instructions and Mechanism of Action

Questions were raised in relation to the content, timing, storage and effectiveness of NRT patches, and the mechanism of action of NRT mouth spray. Combination NRT and smoking while wearing patches were initially understood to be contraindicated by two clients, but counselors were able to elucidate and correct these misconceptions.

#### Physiology of Nicotine Dependence

Two clients expanded on the information given by counselors in relation to nicotine dependence, querying the effect of nicotine from NRT on the brain, and the effect of nicotine from cigarettes on stress.

## Discussion

This is the first qualitative analysis of the content of Quitline smoking cessation counseling sessions. The results show that Quitline counselors collect and deliver a broad range of information, while continually drawing upon clients’ smoking and quit history, and behavioral considerations when personalizing quit plans. All clients at the initial call had made a quit attempt in the past, which is expected given that most people go through multiple attempts and relapse cycles before achieving long-term abstinence (Borland et al., 2012). More importantly, the value of exploring previous quitting experiences allowed for self-reflection by clients so that future quit plans were better informed and improved upon past attempts.

Most clients had used one or more forms of pharmacotherapy prior to their current quit attempt, yet questions regarding pharmacotherapy use and safety were common, and more so for “over the counter” NRT that does not require a prescription or consultation. This highlights the importance of education accompanying pharmacotherapy recommendations, as low adherence and incorrect use (i.e., dose and duration) increases the chances of unsuccessful quit attempts, potentially leading to a perception that pharmacotherapy “doesn’t work” (Mersha et al., 2021). Over-the-counter (OTC) NRT supply does not require health practitioner interaction, side-stepping guidance on optimal use, and may actually reduce the chances of quitting compared with “cold turkey” as shown in one study (Kotz et al., 2014). For this reason, NRT is only publicly subsidized, under the Australian Pharmaceutical Benefits Scheme, when supplied as part of a comprehensive smoking cessation treatment plan that includes counseling (Department of Health, 2022). Conversely, once a person has received information on optimal use when commencing NRT treatment, OTC availability increases the convenience of access for ongoing use beyond initial supply. The addition of an educational and behavioral support program, such as Quitline, allows for an optimal approach to smoking cessation.

Themes and topics found in our analysis closely align with information outlined in Standards 3 (Smoking Cessation Counseling) and 4 (Smoking Cessation Counseling Protocols) of the Australian National Quitline Minimum Standards (NQMS; Cancer Council Victoria, 2021). The NQMS was published in January 2021 following a three-stage development process with an Expert Advisory Group and stakeholders (Cancer Council Victoria, 2021). While the sample of counseling conversations were completed approximately 2 years before the release of these standards, services provided remained consistent in that they generally adhered to the components of the best practice evidence-based smoking cessation protocol, and provided information and advice on stop smoking medications approved by the Therapeutic Goods Administration (TGA). More importantly, despite the diversity in lived experiences within our sample, the NQMS remained applicable. While the aim of this qualitative study was not to audit Quitline practices based on the NQMS, future quantitative research could seek to formally determine adherence to NQMS protocols as a means of quality assurance.

The information provided by Quitline was in line with current evidence and best practice, in that pharmacotherapy was promoted in combination with behavioral support (The Royal Australian College of General Practitioners, 2021; Stead et al., 2016) as well as incorporating important behavior change techniques (BCTs). In an effort to standardize terminology for behavioral interventions in smoking cessation, Michie et al. (2011) developed a taxonomy of 43 BCTs. The themes and topics identified in our analysis were consistent with the majority of BCTs across addressing motivation, maximizing self-regulatory capacity/skills, promoting adjuvant activities, and focusing on the delivery of the intervention, information gathering, and general communication (Michie et al., 2011). More specifically, four BCTs have been identified as significant predictors of smoking cessation in randomized controlled trials when controlling for all BCTs, including commitment, feedback on behavior, social reward, and identity associated with changed behavior (Black et al., 2020). While our analysis did not include prevalence or specific examples of BCTs, further analysis of the data will seek to identify specific examples of content and relational counseling techniques common to both BCT and MI.

Quitline programs offer an accessible option for smoking cessation counseling as observed in the diverse sample population. However, uptake of services in Australia (Australian Institute of Health and Welfare, 2020) and the United States (National Cancer Institute, 2020) tends to be low, and has been trending downward in Australia over the past 10 years in contrast to a steadily increasing uptake in digital interventions (apps and text messaging services; Dono et al., 2022). Cognitive barriers such as stigma (Solomon et al., 2009), as well as misunderstandings about Quitline, variable expectations, and discomfort with the telephone counseling experience have previously been identified as reasons for non-completion of Quitline programs (Albert et al., 2020). Our findings present an example of conventional Quitline support that may assist in elucidating common misconceptions that prevent people from accessing the program. However, uptake of these services ultimately relies on clients’ willingness and capacity to engage in a phone conversation with an unknown counselor and the perceived benefit to their quit attempt. Future research may seek to explore more appealing and accessible platforms, such as smartphone apps, to offer behavioral support that supplements Quitline counseling, or overcomes these barriers for those who choose not to use a phone counseling service.

Our findings may provide a general blueprint for future digital self-help innovations that wish to incorporate a personalized counseling or coaching element, such as chatbots and mHealth applications. Despite the evidence supporting the efficacy of telephonic smoking cessation interventions, they still carry a human resource burden and are often limited in their use. Conversely, digital self-help interventions may be less costly with greater reach, but are generally of lower efficacy (Barnett et al., 2020; Taylor et al., 2017). Real-world examples of personalized behavioral support may help to inform self-help interventions, in turn bridging this gap between efficacy and reach. In Australia, for example, we have observed a steady increase in the use of digital smoking cessation interventions (apps and text messaging services) since 2011, indicating a potential shift in preference for smoking cessation support (Dono et al., 2022). Conversational agents, such as chatbots and virtual assistants, are AI programs that use natural language processing and machine learning to interact with users via audio or text. This technology can emulate human support, and yet while evidence regarding their efficacy for smoking cessation is limited, previous research has found an overall high level of acceptability of conversational agents for smoking cessation (He et al., 2023) and that people interact with and disclose information to chatbots in similar ways they do humans (Ho et al., 2018). Therefore, successfully translating practical examples of smoking cessation counseling into this technology may improve the quality of personalized self-help interventions, and provide highly accessible and potentially comparable options for those who do not want to engage with Quitline.

### Strengths and Limitations

The main strength of this study is the rich source of information derived from real-world Quitline counseling sessions. Responses reflect a practical clinical scenario rather than being elicited for research purposes, thus limiting the risk of the Hawthorne effect from counselors and clients. However, some clients may not have provided counselors with information that was completely true to their experiences or behaviors. In addition, existing guidelines, standards and protocols were not viewed until after inductive coding to minimize the risk of bias in theme and topic development.

There are several limitations due to the exploratory nature of this study, yet it sets an agenda for further analyses of practical smoking cessation counseling delivered outside of the research setting. First, detailed information regarding individual counselors’ level of experience or training was not available. Generalizability of the results may be restricted by geographic location but remain relevant in the Australian context given the consistency in Quitline training and delivery of Quitline counseling services between states and territories. We were unable to follow-up cessation outcomes of clients, determine Quitline program completion rates, or identify specific elements of Quitline counseling sessions that may be predictors of favorable smoking cessation outcomes. The amount of time spent on each topic was not collected, which in combination with smoking cessation outcomes, would allow for future investigation into potential dose-response relationships. However, given there was wide variation in call length, one could argue time spent on topics may be unrelated to smoking cessation outcomes, in that the quality of the counseling is more important than the quantity. Further studies could also explore relationships between level of engagement by clients with Quitline counselors and smoking cessation outcomes or program completion rates. Finally, the sample of Quitline sessions may also introduce selection bias; however, purposive representation of demographic groups, including priority groups, was mostly balanced, and sampling was not completed based on individual dialogue content.

### Implications for Practice

Analyzing real-world Quitline counseling sessions can help to better understand the smoking cessation counseling process as well as client experiences, issues, needs, and expectations. Findings highlight the importance of education alongside pharmacotherapy to improve adherence and smoking cessation outcomes. As digital health capability increases, we need to consider what other delivery modalities can be developed and implemented at scale to reach and support those who prefer not access Quitline.

## Conclusion

In this first independent qualitative analysis of Quitline counseling sessions in Australia, we found that counselors deliver comprehensive and evidence-based intervention, tailored to the individual client. In contrast, client questions centered around understanding the practicalities and safety of pharmacotherapy. Findings may help to improve current practice, enhance self-help interventions and advise future innovations, particularly for people who do not want to engage with Quitline. Further investigation into the use of BCTs within Quitline conversations will provide a more detailed practical insight into behavioral support for smoking cessation.

## Supplemental Material

sj-docx-1-heb-10.1177_10901981231206068 – Supplemental material for Smoking Cessation Counseling in Practice: A Qualitative Analysis of Quitline Conversations in Queensland, Australia Click here for additional data file. Supplemental material, sj-docx-1-heb-10.1177_10901981231206068 for Smoking Cessation Counseling in Practice: A Qualitative Analysis of Quitline Conversations in Queensland, Australia by Hollie Bendotti, Sheleigh Lawler, Coral Gartner, David Ireland and Henry M. Marshall in Health Education & Behavior

sj-docx-2-heb-10.1177_10901981231206068 – Supplemental material for Smoking Cessation Counseling in Practice: A Qualitative Analysis of Quitline Conversations in Queensland, Australia Click here for additional data file. Supplemental material, sj-docx-2-heb-10.1177_10901981231206068 for Smoking Cessation Counseling in Practice: A Qualitative Analysis of Quitline Conversations in Queensland, Australia by Hollie Bendotti, Sheleigh Lawler, Coral Gartner, David Ireland and Henry M. Marshall in Health Education & Behavior
