# Supplementary material for: Smoking Cessation Counseling in Practice: A Qualitative Analysis of Quitline Conversations in Queensland, Australia
Source: Health Educ Behav. 2023 Oct 17;51(1):43–53. doi: 10.1177/10901981231206068 (PMC10785561; doi:10.1177/10901981231206068)
Supplement: sj-docx-1-heb-10.1177_10901981231206068 – Supplemental material for Smoking Cessation Counseling in Practice: A Qualitative Analysis of Quitline Conversations in Queensland, Australia [file sj-docx-1-heb-10.1177_10901981231206068.docx]

**Supplementary Table 1: Summary of client demographics, smoking behaviour, quit history and quit plans.**

| **ID** | **Type of Call** | **Type of initiation** | **Referred from** | **Length (mins)** | **Sex** | **Age** | **Location** | **Aboriginal and/or Torres Strait Islander** | **Smoking status** | **Length current quit** | **Current method** | **Cigarettes per day (post-attempt)** | **Cigarettes per day (pre-quit)** | **Age started smoking** | **Years smoking** | **Previous quit attempt/s** | **Length longest quit** | **Method/s previous quit attempt/s** | **Follow-up discussed** | **Quit date set** | **Pharmacotherapy discussed** |
| --- | --- | --- | --- | --- | --- | --- | --- | --- | --- | --- | --- | --- | --- | --- | --- | --- | --- | --- | --- | --- | --- |
| **1** | Initial | Proactive | Hospital | 50:37 | F | . | Regional/  Rural | Y | Current | . |  | . | 30.0 | 7 | . | Y | 6 months | Cold turkey | Y | N | Combo NRT |
| **2** | Initial | Reactive | . | 38:44 | F | 60 | Metropolitan | N | Current | . | . | . | 8.5^a^ | 16 | 44 | Y | 1 week | Cold turkey | N | Y | Varenicline |
| **3** | Initial | Proactive | Hospital | 29:59 | F | 63 | Regional/  Rural | . | Current | . | . | . | 15.0 | 19 | 44 | Y | 3 days | NRT patch | Y | Y | Combo NRT |
| **4** | Initial | Proactive | Dentist | 43:50 | M | 49 | Metropolitan | . | Current | . | . | . | 10.0 | 13 | 36 | Y | 18 months | NRT patch | N | Y | Combo NRT |
| **5** | Initial | Reactive | . | 44:28 | M | 66 | Regional/  Rural | N | Current | . | . | . | 27.5^a^ | 28 | 38 | Y | 2 weeks | NRT lozenge | Y | N | Combo NRT |
| **6** | Initial | Proactive | Work | 21:12 | M | 54 | Metropolitan | . | Current | . | . | . | 30.0 | 14 | 40 | Y | 0 days | Varenicline; NRT patch; NRT gum | Y | N | NRT patch |
| **7** | Initial | Proactive | Work | 30:22 | M | 33 | Regional/  Rural | . | Current | . | . | . | 7.0 | 18 | 15 | Y | 12 weeks | Patch; Varenicline; Cold turkey | Y | Y | Oral NRT (gum) |
| **8** | Support | Proactive | . | 34:05 | F | 26 | Regional/  Rural | Y | Quit | 3 days | NRT patch, NRT gum | . | 15.0 | . | . | Y | . | Cold turkey | Y | NA | Combo NRT |
| **9** | Initial | Proactive | Community mental health | 18:13 | F | 33 | Regional/  Rural | . | Current | . | . | . | 25.0^a^ | 12 | 21 | Y | 4 years | Varenicline; NRT patch | Y | Y | Combo NRT |
| **10** | Support | Proactive | . | 39:15 | M | 60 | Regional/  Rural | . | Current; cutting down | . | NRT patch, NRT gum | 10 | 13.0 | 15 | 45 | Y | 10 months | . | N^b^ | N^a^ | Oral NRT (gum) |
| **11** | Initial | Proactive | Hospital | 40:46 | M | 52 | Regional/  Rural | . | Current | . | . | . | 10.5^a^ | 13 | 39 | Y | 10 days | Varenicline; NRT patch | Y | N | Oral NRT (spray, lozenges) |
| **12** | Support | Proactive | . | 21:23 | M | 61 | Metropolitan | . | Current | 2 days | NRT patch | 12 | . | . | . | . | . | NRT patch; NRT gum | Y | N | Combo NRT |
| **13** | Initial | Proactive | Hospital | 32:11 | F | 48 | Regional/  Rural | N | Current | . | . | . | 11.0^a^ | . | . | Y | . | Cold turkey | Y | N | Combo NRT |
| **14** | Initial | Proactive | Coach nurse | 23:35 | F | 61 | Metropolitan | . | Quit | 2 weeks | Varenicline | 0 | 10.0 | . | . | Y | 1 month | Varenicline | Y | NA | Varenicline |
| **15** | Initial | Reactive | Drug & alcohol counselling | 31:57 | M | 29 | Metropolitan | N | Current | . | . |  | 17.5^a^ | 14 | 15 | Y | 3 days | NRT patch; NRT gum | N | N | Combo NRT |
| **16** | Initial | Proactive | . | 31:51 | M | 26 | Regional/  Rural | . | Current | . | . | . | 17.5^a^ | 20 | 6 | Y | 3 months | Cold turkey | Y | N | Combo NRT |
| **17** | Initial | Proactive | . | 49:50 | F | 40 | Regional/  Rural | N | Current | . | . | . | 17.5^a^ | 14 | 26 | Y | . | NRT patch | Y | Y | Combo NRT |
| **18** | Initial | Proactive | Dentist | 31:04 | F | 28 | Regional/  Rural | Y | Current | . | . | . | 10.0 | 17 | 11 | Y | 18 months | Varenicline; NRT patch; NRT gum | Y | N | Combo NRT |
| **19** | Initial | Proactive | . | 37:10 | F | 37 | Regional/  Rural | N | Current | . | . | . | 15.0 | 17 | 20 | Y | 6 weeks | Cold turkey | Y | N | Combo NRT |
| **20** | Initial | Proactive | Self | 47:25 | F | 19 | Metropolitan | . | Current | . | . | . | 7.0 | 18 | 1 | Y | 4 days | Cold turkey | Y | N | Combo NRT |
| **21** | Initial | Proactive | . | 34:56 | F | 47 | Regional/  Rural | . | Current; cutting down | . | Buproprion | 3 | 20.0 | 15 | 32 | Y | 3 months | Buproprion | Y | Y | Buproprion; Oral NRT (lozenges) |
| **22** | Support | Proactive | . | 15:59 | F | 41 | Metropolitan | . | Current; cutting down | 3 days | NRT patch, NRT gum | 0^c^ | . | . | . | . | . | . | N^d^ | N | Combo NRT |
| **23** | Initial | Proactive | Work | 33:50 | M | 35 | Regional/  Rural | . | Current | . | . | . | 20.0 | . | . | Y | 5 weeks | Varenicline; NRT patch; NRT lozenge | Y | Y | Combo NRT |
| **24** | Initial | Proactive | . | 34:33 | F | 44 | Metropolitan | . | Current | . | . | . | 15.0 | 19 | 25 | Y | . | Varenicline; NRT gum | Y | N | Combo NRT |
| **25** | Initial | Reactive | . | 46:30 | M | 38 | Regional/  Rural | N | Current | . | . | . | . | 16 | 22 | Y | 5 weeks | Varenicline; Buproprion | Y | N | Combo NRT |
| **26** | Initial | Proactive | . | 33:51 | M | 32 | Metropolitan | . | Current | . | . | . | 17.5^a^ | . | . | Y | 2 years | Cold turkey; Varenicline | Y | N | Combo NRT |
| **27** | Initial | Proactive | . | 56:18 | F | 65 | Regional/  Rural | . | Current | . | NRT patch, NRT gum | . | 20.0 | 16 | 49 | Y | 5 years | Cold turkey | Y | N | Combo NRT |
| **28** | Initial | Proactive | . | 38:26 | M | 49 | Regional/  Rural | N | Current | . | . | . | 27.5 | 14 | 35 | Y | 2 years | Laser acupuncture | Y | Y | Combo NRT |
| **29** | Initial | Proactive | Work | 52:54 | M | 51 | Regional/  Rural | . | Quit | 3 weeks | Varenicline | 0 | 20.0 | 18 | 33 | Y | 4 weeks | Cold turkey; NRT gum; Varenicline | Y | NA | Varenicline |
| **30** | Initial | Proactive | Hospital | 34:21 | F | 37 | Metropolitan | N | Quit | 1 week | NRT patch | 0 | 10.0 | 20 | 17 | Y | 18 months | . | Y | NA | NRT patch |

^a.^Average number based on range given

^b.^Incomplete audio file

^c.^ <1 cigarette per day

^d.^ Final call of Quitline program
