# Supplementary material for: Smoking Cessation Counseling in Practice: A Qualitative Analysis of Quitline Conversations in Queensland, Australia
Source: Health Educ Behav. 2023 Oct 17;51(1):43–53. doi: 10.1177/10901981231206068 (PMC10785561; doi:10.1177/10901981231206068)
Supplement: sj-docx-2-heb-10.1177_10901981231206068 – Supplemental material for Smoking Cessation Counseling in Practice: A Qualitative Analysis of Quitline Conversations in Queensland, Australia [file sj-docx-2-heb-10.1177_10901981231206068.docx]

**Supplementary Table 2: Description of conversation themes and topics**

| **Main theme** | **Topics** | **Sub-topics** | **Description** |
| --- | --- | --- | --- |
| Client details and building rapport | Introduction | *Referral source* | Greetings, confirmation of referral, and consent to proceed. |
|  | Demographic information | *Offer of Indigenous counsellor* | Date of birth Postcode, Indigenous status. |
|  | Personal life |  | General conversation about client's personal life. |
| Client history and motivation to quit | Benefits of quitting | *Financial* | Discussions concerning anticipated and felt benefits of quitting including monetary savings relative to individual smoking behaviour, health improvements expected after quitting smoking, and social benefits relative to family and relationships. |
|  |  | *Health* |  |
|  |  | *Social* |  |
|  | Cold turkey |  | Previous experiences and difficulty of quitting without support. |
|  | Current quit attempt |  | How clients are tracking following recent quit attempt including changes in smoking behaviour, adherence to quit plan and pharmacotherapy, issues and feelings. |
|  | Health concerns & effects of smoking |  | Health issues, concerns or deterioration in health conditions due to smoking. |
|  | Importance of quitting |  | Gauging and reflecting on why quitting smoking is important for client. |
|  | Motivation for quitting |  | Exploring personal reasons and motivations for wanting to quit smoking. |
|  | Other health problems |  | Discussions about client’s health problems outside of the context of smoking. |
|  | Physical feelings and withdrawals |  | Discussing clients' current or past experience with withdrawals and feelings when going without a cigarette. |
|  | Pregnancy |  | Counsellor education and discussion about smoking and pregnancy. |
|  | Smoking & quit history | *Relapse* | Exploring number of years smoking, reasons for starting smoking, changes in smoking behaviour, previous experience with pharmacotherapies and quit attempts in general including reasons fort relapse. |
|  | Nicotine dependence assessment |  | Determining level of nicotine dependence (number of cigarettes per day, time to first cigarette) with/without assessment results and agreement from client. |
| Pharmacotherapy | Nicotine replacement therapy (NRT) & Combination NRT |  | Counsellor education on NRT mechanism of action and promotion of combination nicotine replacement therapy. |
|  | NRT patches | *NRT patch instructions* | Counsellor education about the treatment course, dosage, timing and placement of NRT patches. May or may not be personalised for specific levels of dependence and health issues. |
|  |  | *NRT patch issues/side effects* | Counsellor education about the mechanism of action of NRT patches, including potential side effects and associated solutions. |
|  | NRT safety considerations |  | Standard NRT safety questions regarding cardiac problems in the last 24hrs, pregnancy and skin conditions. Asked prior to dispensing of products. |
|  | Oral NRT | *Oral NRT side-effects* | Counsellor education about the purpose and mechanism of action of oral NRT products, including potential side effects and associated solutions. |
|  |  | *Oral NRT instructions* | Counsellor education about the dosage, timing and instructions for use of Oral NRT products including gum, lozenges, spray and inhalers. |
|  | Pharmaceutical Benefits Scheme |  | Counsellor education about reduced cost of NRT products through the Pharmaceutical Benefits Scheme via prescription from GP. |
|  | Prescription pharmacotherapy | *Bupropion/instructions* | Counsellor education about the purpose and mechanism of action of prescription pharmacotherapies such as varenicline and bupropion, including potential side effects, instructions for treatment course length, and access through GP. |
|  |  | *Varenicline/side-effects/instructions* |  |
| Behavioural aspects of quitting and relationship with smoking | Routines, triggers and strategies | *Routines* | Routines and triggers identified by client/counsellor and associated management strategies discussed. |
|  |  | *Triggers* |  |
|  |  | *Strategies* |  |
|  | Confidence to quit |  | Questions to gauge clients' level of confidence and identify strategies/tools to improve low confidence. |
|  | Smoker identity |  | Understanding attitudes towards and personal relationship with smoking. |
| Understanding nicotine dependence and other important considerations | Other medications, alcohol and coffee |  | Counsellor education about reducing intake of alcohol and coffee when quitting smoking, as well as notifying their GP to review medication dosages following quit attempt. |
|  | Physical vs behavioural sides of addiction |  | Counsellor education about the need to manage the physical and behavioural aspects of quitting smoking. |
|  | Physiology of nicotine dependence |  | Counsellor education about the physiology of nicotine dependence including how it influences cravings and relapse, and time required to deactivate nicotine receptors. |
| Additional support and smoking cessation resources | Additional resources | *Hypnosis* | Other resources, tools or treatments use by clients or promoted by counsellors. |
|  |  | *Laser acupuncture* |  |
|  |  | *Vaping* |  |
|  |  | *Apps* |  |
|  | Internal referral to other health services |  | Offer of referral to partner public health services for people with specific health conditions that make them eligible for additional smoking cessation support. |
|  | Professional support |  | Other professional support services available including GP, carers support and mental health services. |
|  | Social support |  | Identifying people that will be of support for a clients' quit attempt. |
| Planning, goal setting and follow up | Planning to quit & goal setting | *Setting quit date* | Goals relative to quit attempt, health, or personal life. Planning quit attempt including setting quit date, type of plan, personal rewards, and addressing concerns about weight gain. |
|  |  | *Rewards* |  |
|  |  | *Cutting down but not quitting* |  |
|  |  | *Weight concerns* |  |
|  | Quitline program | *Eligibility for types of programs* | Elements of the Quitline program discussed including description of services available to clients', organising NRT products and further information packs, tailoring products not normally included in the program if required, and next steps following the completion of the Quitline program. |
|  |  | *Organising products* |  |
|  |  | *Tailoring products* |  |
|  |  | *Organising welcome pack* |  |
|  |  | *NRT supply follow-up* |  |
|  |  | *End of Quitline program* |  |
|  | Reflection & feedback |  | Counsellor eliciting clients' feedback on their call and encouraging them to reflect upon the most helpful elements for their quit attempt. |
|  | Reinforce quit plan |  | Counsellor repeating agreed upon elements of quit plan including pharmacotherapy schedule, behavioural strategies, and timing of support calls. |
|  | Scheduling follow-up |  | Determining an appropriate day and time for follow-up calls, or advising the client to expect a call in approximately 2 weeks time. |
